# The Updated Phylogenies of the Phasianidae Based on Combined Data of Nuclear and Mitochondrial DNA

**DOI:** 10.1371/journal.pone.0095786

**Published:** 2014-04-18

**Authors:** Yong-Yi Shen, Kun Dai, Xue Cao, Robert W. Murphy, Xue-Juan Shen, Ya-Ping Zhang

**Affiliations:** 1 State Key Laboratory of Genetic Resources and Evolution, Kunming Institute of Zoology, the Chinese Academy of Sciences, Kunming, China; 2 Joint Influenza Research Centre (SUMC/HKU), Shantou University Medical College, Shantou, China; 3 Xinjiang Institute of Ecology and Geography, the Chinese Academy of Sciences, Urumqi, China; 4 Department of Natural History, Royal Ontario Museum, Toronto, Canada; 5 Laboratory for Conservation and Utilization of Bio-resources, Yunnan University, Kunming, China; 6 University of the Chinese Academy of Sciences, Beijing, China; University of Texas Health Science Center at San Antonio, United States of America

## Abstract

The phylogenetic relationships of species in the Phasianidae, Order Galliformes, are the object of intensive study. However, convergent morphological evolution and rapid species radiation result in much ambiguity in the group. Further, matrilineal (mtDNA) genealogies conflict with trees based on nuclear DNA retrotransposable elements. Herein, we analyze 39 nearly complete mitochondrial genomes (three new) and up to seven nuclear DNA segments. We combine these multiple unlinked, more informative genetic markers to infer historical relationships of the major groups of phasianids. The nuclear DNA tree is largely congruent with the tree derived from mt genomes. However, branching orders of mt/nuclear trees largely conflict with those based on retrotransposons. For example, *Gallus/Bambusicola/Francolinus* forms the sister-group of *Coturnix/Alectoris* in the nuclear/mtDNA trees, yet the tree based on retrotransposable elements roots the former at the base of the tree and not with the latter. Further, while peafowls cluster with *Gallus/Coturnix* in the mt tree, they root at the base of the phasianids following *Gallus* in the tree based on retrotransposable elements. The conflicting branch orders in nuclear/mtDNA and retrotransposons-based trees in our study reveal the complex topology of the Phasianidae.

## Introduction

Rapid species radiations often result in ambiguous phylogenetic relationships because too little time is available to accrue and fix shared derived character states. This may manifest itself, in part, as incomplete lineage sorting. In such cases, different datasets, such as genes, may resolve conflicting suites of relationships. The Phasianidae, one of four families in the Galliformes, typifies this problem. Rapid radiation and convergent morphological evolution confound the resolution of relationships for many pheasants and partridges. Although the family has been the target of much phylogenetic research [1]–[17], not surprisingly, many unsolved nodes and much conflict remain.

**Figure 1:**
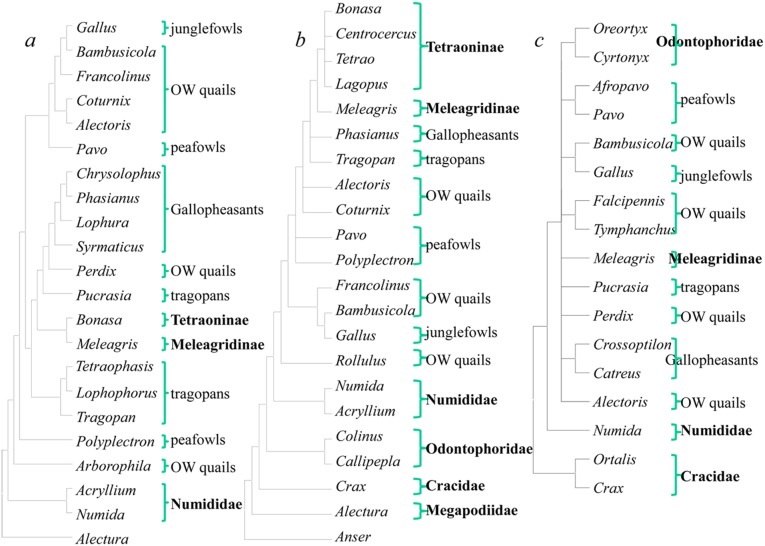
Phylogenetic hypotheses from the mitochondrial (mt) genome and retrotransposable elements for the Phasianidae. (a) Topology based on mt genomes (Shen *et al*. 2010); (b) topology based on insertion events of CR1 retrotransposable nuclear DNA elements [6], [17], [20]; (C) topology based on nuclear DNA segments (Crowe et al., 2006).

Most previous molecular studies of phasianids analyze either one or a few mitochondrial (mt) genes [3], [9], [10], [12], a single nuclear gene [1], [18], or a combination of mt and a few nuclear gene sequences [2], [7]. Employing complete mt genomes, Shen et al. [19] resolve a well-supported topology. The topology (Figure 1A) is largely congruent with previous molecular studies based on mt genes and nuclear segments [2], [7]. However, this tree strongly conflicts with that based on retrotransposable elements (Figure 1B) [6], [17], [20]. For example, in the matrilineal genealogy, *Gallus* forms the sister-group of *Coturnix*, while the tree based on retrotransposable elements roots *Gallus* at the base of the phasianids, and *Coturnix* is the sister-group of the gallopheasants. Further, while *Pavo* is the sister-group of *Gallus/Coturnix* in the mt tree, it roots at the base of the phasianids following *Gallus* in the tree based on retrotransposable elements. Trees based on unclear genes exhibit many ambiguous nodes (Figure 1C).

The branching order in trees based on the mt genome conflicts with those derived from nuclear retrotransposons. This incongruence requires a reassessment of the phylogeny of the Phasianidae. Mitochondrial DNA markers reflect the matrilineal genealogy only; they do not provide information on paternal contributions. For retrotransposons, only a few phylogenetic approaches use indels as characters. Few genomes are available from which to design conservative retrotransposon primers for phasianids; only the chicken and a limited number of other avian genomes are available. This situation makes it difficult to obtain a sufficient number of phylogenetically informative characters. Considering the shortcomings of the mt and retrotransposon approaches, herein we report the sequencing of up to seven independent nuclear segments for 20 species and the complete mt genomes of three phasianids. We obtain other mt genomes from GenBank (Table S1 in File S1) and then combine these unlinked markers for the major groups of the Phasianidae to infer phylogenetic relationships.

## Materials and Methods

### Specimens Sampling

The Animal Use Ethics Committee of the Kunming Institute of Zoology, the Chinese Academy of Sciences approved the study. *Argusianus argus*, *Crossoptilon crossoptilon* and *Ithaginis cruentus* were used for mt genome sequencing. A total of 23 species were used for nuclear gene sequencing (Table S1 File S1). Feather samples of *Argusianus argus* were provided by Beijing Zoo and the Museum of the Kunming Institute of Zoology provided muscle tissue for all other samples. Additional complete mt genomes and nuclear segments were obtained from GenBank (Table S1 in File S1).

### DNA Extraction, PCR Amplification, and Sequencing

Total genomic DNA was extracted using standard 3-step phenol/chloroform extraction methods [21]. For mitochondrial genomes, primers were described in our previous study [19]. For seven nuclear segments (*BDNF*, *CMOS*, *FIB4*, *NGFB*, *NTF3*, *OVOG*, and *ZENK*), primers were described in Table S2 in File S1. PCR amplifications were conducted in a 50 µl volume containing 5 µl of 10×reaction buffer, 0.2 mM dNTPs, 0.2 µM each primer, 1.5 U Taq DNA polymerase (TaKaRa Biosystems), and approximately 2 ng total DNA. PCR amplifications were carried out using the following parameters: 95°C 4 min, 20 cycles of denaturation at 94°C for 1 min, annealing at 60–50°C (1 min; 0.5°C/cycle), extension at 72°C for 1 min, and finally 15 cycles of 94°C 1 min, 50°C 1 min, 72°C 1 min. PCR products were cleaned using Watson RCR Purification Kits (Watson BioTechnologies, Shanghai). PCR products were sequenced at least three times in both directions on an ABI 3730 Sequencer (Applied Biosystems, Foster, CA, USA) using the ABI PRISM BigDye Terminator v3.0 sequencing kit. DNA sequences were edited using DNAstar Seqman software (DNASTAR Inc., Madison, WI, USA). The newly determined sequences were deposited in GenBank (GenBank accession numbers: JQ713766–JQ713768; JQ713656–JQ713765).

### Phylogenetic Reconstruction

The nucleotide sequence data sets were initially aligned using ClustalX 1.81 [22] with default parameters. The combined and individual 13 mitochondrial protein coding genes, and the combined data of seven nuclear segments were analyzed separately using maximum likelihood (ML) implemented in PAUP* 4.0b10 [23]. Modeltest 3.7 [24] was used to select the preferred models of evolution under the Akaike Information Criterion. ML heuristic searches used TBR branch swapping executed in 100 replicates with the selected models. Because heuristic searches in PAUP* were very slow, we used two additional fast ML-based inference packages using 1,000 replicates each: RAxML [25] and PHYML [26]. Because their topologies were identical, and only a few bootstrap values slightly differed, we only presented trees with bootstrap values from PAUP*. Bayesian inference (BI) was performed using MrBayes 3.1.2 [27]. The analyses used models estimated with Modeltest 3.7 under AIC. Two separate runs were performed with four Markov chains. Each run was conducted with 3×10^6^ generations and sampled every 100 generations. When the log-likelihood scores were found to stabilize, a consensus tree was calculated after omitting the first 25% trees as burn-in. In all these topology reconstruction, *Alectura lathami* was set as the outgroup according a previous study [19].

## Results

### Phylogenetic Analyses of the Mitochondrial DNA Dataset

**Figure 2:**
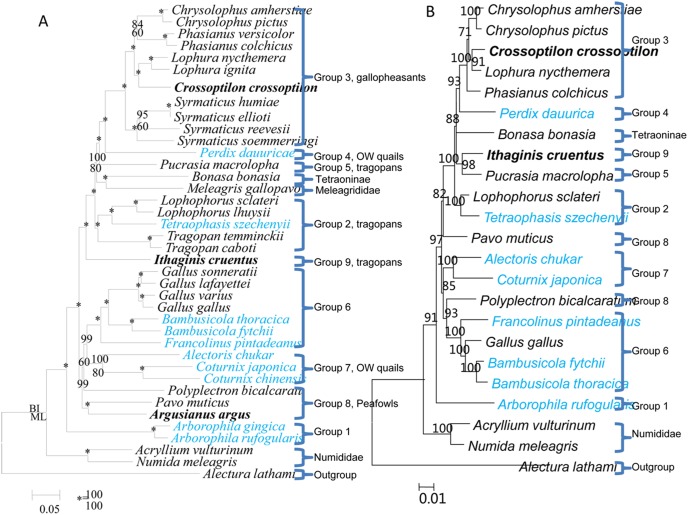
Phylogenetic hypotheses based on the complete mitochondrial genomes and nuclear segments for the Phasianidae. (a) Mt genomes; newly sequenced mt genomes denoted in bold. (b) Nuclear segments. Bayesian posterior probabilities >70%, and maximum likelihood bootstrap proportions >50% were indicated on the branches.

We evaluated 39 mt genomes including those from GenBank. The 13 protein-coding genes consisted of 11,359 aligned nucleotide positions and the best-fit model of evolution was GTR+I+G. ML and BI analyses involving equal weight for each position resolved a single, robust tree (Figure 2A). Eight lineages, each with very high BSPs and BPPs, were resolved as follows: Group 1, *Arborophila*; Group 2, *Tragopan*, *Lophophorus*, and *Tetraophasis*; Group 3, *Chrysolophus*, *Phasianus*, *Lophura*, and *Syrmaticus*; Group 4, *Perdix*; Group 5, *Pucrasia*; Group 6, *Gallus*, *Bambusicola*, and *Francolinus*; Group 7, *Coturnix* and *Alectoris*; and Group 8, the peacocks.

Three additional weighting strategies were applied to the analysis of combined 13 protein-coding genes to avoid possible bias of nucleotide composition and saturation: (1) excluding the 3^rd^ codon positions (Figure S1), (2) recoding the 3^rd^ codon position nucleotides to two-state categories, R (purine) and Y (pyrimidine) (Figure S2), and (3) recoding the 1^st^ and the 3^rd^ codon position nucleotides to RY categories (Figure S3). The major topologies based on these weighting strategies were the same as evaluating all positions equally (Figure 2A).

Individual mt gene trees (Figure S4) were largely congruent with the mt genomic tree. As expected because of a low number of potentially phylogenetically informative characters for individual genes, nodes were supported by lower BSPs.

### Phylogenetic Analyses of Nuclear Dataset

Segments of seven nuclear genes (*BDNF*, *CMOS*, *FIB4*, *NGFB*, *NTF3*, *OVOG*, and *ZENK*) were sequenced (Table S1 in File S1). Combined, these data consisted of 4,604 nucleotide positions. The best-fit model of nucleotide substitution was TrN+I+G. ML and BI analyses of the combined data resolved a single tree (Figure 2B). The nuclear tree was largely congruent with mitochondrial tree although some nodes conflicted. For example, *Ithagins cruentus* rooted at the base of Gallopheasants/tragopans in mt tree but the nuclear tree resolved it as the sister group of *Pucrasia*. The position of peafowls was unstable in nuclear tree, while in mt tree they rooted at the base of *Gallus*/Old World quails.

### Phylogenetic Analyses of the Combined Mitochondrial and Nuclear DNA Dataset

The mt and nuclear datasets shared 23 species. The combined dataset consisted of 15,972 aligned nucleotide positions. The best-fit model of nucleotide substitution was GTR+I+G. The topology (Figure S5) based on the combined dataset was nearly identical to that of mt tree (Figure 2A); they differed in the position of *Phasianus*. The mt genome tree clustered *Phasianus* with *Chrysolophus* with low support values (BPP = 84, and BSP = 60). In turn, this group clustered as the sister group of *Lophura/Crossoptilon* (BPP = 100, and BSP = 100). Trees based on the combined dataset clusterd *Chrysolophus* with *Lophura/Crossoptilon* (BPP = 99), then with *Phasianus* (BPP = 100). Trees derived from combined dataset were largely congruent with the nuclear gene phylogeny. However, a few conflicts occurred. For example, the nuclear gene tree resolved *Ithagins* and *Pucrasia* as sister taxa, while in combined and mt genome trees separated them far apart.

## Discussion

Our mt genome tree depicts *Arborophila* as the sister-group to all other phasianids plus the Meleagrididae and Tetraonidae. *Tetraophasis* clusters independently with *Lophophorus* and their sister-group is *Tragopan*; *Perdix* and *Arborophila* do not cluster with other partridges. The non-monophyly of the pheasants and partridges is more common than not and this resolution involves a strongly supported association of *Gallus*, *Bambusicola*, and *Francolinus*. The previous matrilineal genealogy did not cluster *Polyplectron* with *Pavo* [16], [19]. Herein, we add a new peacock–*Argusianus argus*. *Polyplectron* forms the sister-group of *Gallus*/*Bambusicola*/*Coturnix* albeit with relatively low support. This group clusters with other peacocks (*Pavo* and *Argusianus*). The phylogenetic position of *Polyplectron* remains unstable. More data involving new peacocks may further resolve this group’s position.

We add the nearly complete mt genome of *Crossoptilon crossoptilon* and analyses involving this species clusters it with *Lophura* (BPP = 100; BSP = 100). Analyses of the new mt genome of *Ithaginis cruentus* strongly unites it with the gallopheasants/*Perdix*/tragopans/Tetraoninae/Meleagrididae (BPP = 100; BSP = 100). *Tragopan*, *Ithaginis, Pucrasia, Lophophorus* form the tragopans tribe [28]. Our resolution of a sister relationship for *Tragopan* and *Lophophorus* is well supported, but *Ithaginis* and *Pucrasia* do not cluster with them. The mt genome tree also does not support the morphological and behavioral placement of *Ithaginis* as the sister-group of New World and Old World quails [4]. Previous molecular studies did not conclusively resolve the phyletic position of *Ithaginis* [2], [10].

The mt genomes provide a greater abundance of information, thus, have a greater likelihood of fully resolving a tree than individual protein-coding genes (Figure S4). In our analyses, almost all nodes receive very high support. Thus, the rapid rate of mutation renders the mt genome phylogenetically informative at the levels of genera and species for the phasianids.

Reliance on mt data for phylogenetic reconstruction may be fraught with problems. Functionally, in most animals the mt genome serves as a single, large genetic locus and it provides a matrilineal perspective only on the evolutionary history of a group [29], [30]. Paternal contributions are not considered. Thus, mtDNA data alone are often inadequate for macroevolutionary phylogenetic analyses, especially in the face of complex evolutionary scenarios such as gene introgression, hybridization, and/or selection [31]. Our seven nuclear segments address this concern. BSPs tend to be lower in nuclear tree compared to the mt tree. The relatively slow rate of mutation rate of nuclear DNA compared to mtDNA generally results in relatively poorly resolved nuclear gene trees.

Previous studies based on a single nuclear gene failed to solve many nodes [1], [18]. In contrast, our multi-gene analyses resolve many nodes with very high levels of support. This result indicates that additional informative sites greatly help to resolve ambiguous relationships. Our nuclear phylogeny is largely congruent with trees derived from mt genomes. However, our nuclear tree resolves *Ithagins* and *Pucrasia* as sister groups, but the mt tree depicts divergent relationships. The position of *Ithagins* was ambiguous in the previous morphological-behavioral parsimony cladogram of Dyke et al. (2003) and molecular studies [2], [9], [10]. The position of *Ithagins* received high BSPs in our mt and nuclear trees, yet further explorations into the nature of conflicting trees is necessary. The positions of peafowl are unstable in nuclear tree. *Pavo* and *Polyplectron* do not cluster together, including in our mt tree.

The tree based on combined mitochondrial and nuclear dataset results a well-supported tree (Figure S5). In mt genome tree (Figure 2A), the position of genus *Phasianus* is not well supported; it clusters with *Chrysolophus* with low support values (BPP = 84; BSP = 60). In contrast, the trees of the combined datasets cluster *Phasianus* with (*Chrysolophus*, (*Lophura,Crossoptilon*), and with strong support. The positions of peafowls are unstable in the mt genome, nuclear data, and combined data trees. More species of peafowl and additional markers may resolve the position of the peafowl.

Retrotransposons-based trees (Figure 1B) strongly conflict with nuclear/mtDNA trees (Figure 2) at the level of genus. For example, in the latter case, *Gallus/Bambusicola/Francolinus* forms the sister-group of *Coturnix/Alectoris*, while the former analyses root *Gallus/Bambusicola/Francolinus* at the base of the phasianids, and *Coturnix/Alectoris* formed the sister-group of gallopheasants/tragopans. Further, while peafowls cluster with *Gallus/Coturnix* in the mt tree, they root at the base of the phasianids following *Gallus* in the tree based on retrotransposable elements.

Retrotransposon data often consist of insertion/deletion (indel) events. Only a few phylogenetic approaches use indels as characters. Most researchers either delete them or treat the gaps as missing data. Indels cannot resolve relationships of clades branching off the focal clade–the lineage leading to the species in which the markers are originally identified–either before or after the insertion event [6]. Further, few genomes are available for identifying retrotransposon markers. These consist of the chicken and a limited number of other avian genomes. This paucity not only limits the design of conservative PCR primers for the target group, it also limits identification of an adequate number of informative characters. Conclusions based upon a few markers may be lead to inaccurate findings [17], [32]. Thus, retrotransposons appear to be severely limited in their ability to resolve relationships at the hierarchical levels of genus and species, especially in cases of rapid radiations of species. This may explain the conflicting branching orders.

In conclusion, we combine mt genomes and segments of seven nuclear genes to reassess the phylogenetic relationships of phasianids. These multiple unlinked and informative genetic markers provide an updated topology. Our nuclear gene phylogeny is largely congruent with trees derived from mt genomes. However, our mt and nuclear topology largely conflict retrotransposons-based trees.

## Supporting Information

Figure S1 **Bayesian inference analyses of 13 mt genes that excluding the 3^rd^ codon position.**
(TIF)

Figure S2 **Bayesian inference analyses of 13 mt genes that recoding the 3^rd^ codon position nucleotides to two-state categories, R (purine) and Y (pyrimidine).**
(TIF)

Figure S3 **Bayesian inference analyses of 13 mt genes that recoding the 1^st^ and the 3^rd^ codon position nucleotides to two-state categories, R (purine) and Y (pyrimidine).**
(TIF)

Figure S4 **Bayesian inference analyses of individual mt genes and control region (CR).** Each run was conducted with 5,000,000 generations and sampled every 100 generations. Bayesian Posterior Probabilities >70% were indicated on the branches. (A) *12S*, 1,036 aligned sites; (B) *16S*, 1,702 aligned sites;(C) *ATP6*, 681 aligned sites; (D) *ATP8*, 165 aligned sites; (E) *CoxI*, 1,548 aligned sites; (F) *CoxII*, 681 aligned sites; (G) *CoxIII*, 783 aligned sites; (H) CR, 1,352 aligned sites; (I) *ND1*, 972 aligned sites; (J) *ND2*, 1,038 aligned sites; (K) *ND3*, 348 aligned sites; (L) *ND4*, 1,377 aligned sites; (M) *ND4L*, 291 aligned sites; (N) *ND5*, 1,818 aligned sites; (O) *ND6*, 519 aligned sites; (P) *CytB*, 1,137 aligned sites.
(PDF)

Figure S5 Bayesian phylogenetic tree based on the combined dataset of 13 mt protein-coding genes and seven nuclear segments.
(TIF)

File S1 **This file contains Table S1 and Table S2.** Table S1, Source of sequence data for mitochondrial genomes and nuclear segments. Table S2, List of primers used in this study of the Phasianidae.
(DOCX)
