# Supplementary figures and images for: The Updated Phylogenies of the Phasianidae Based on Combined Data of Nuclear and Mitochondrial DNA
Source: PLoS One. 2014 Apr 18;9(4):e95786. doi: 10.1371/journal.pone.0095786 (PMC3991718; doi:10.1371/journal.pone.0095786)

(A): 12S

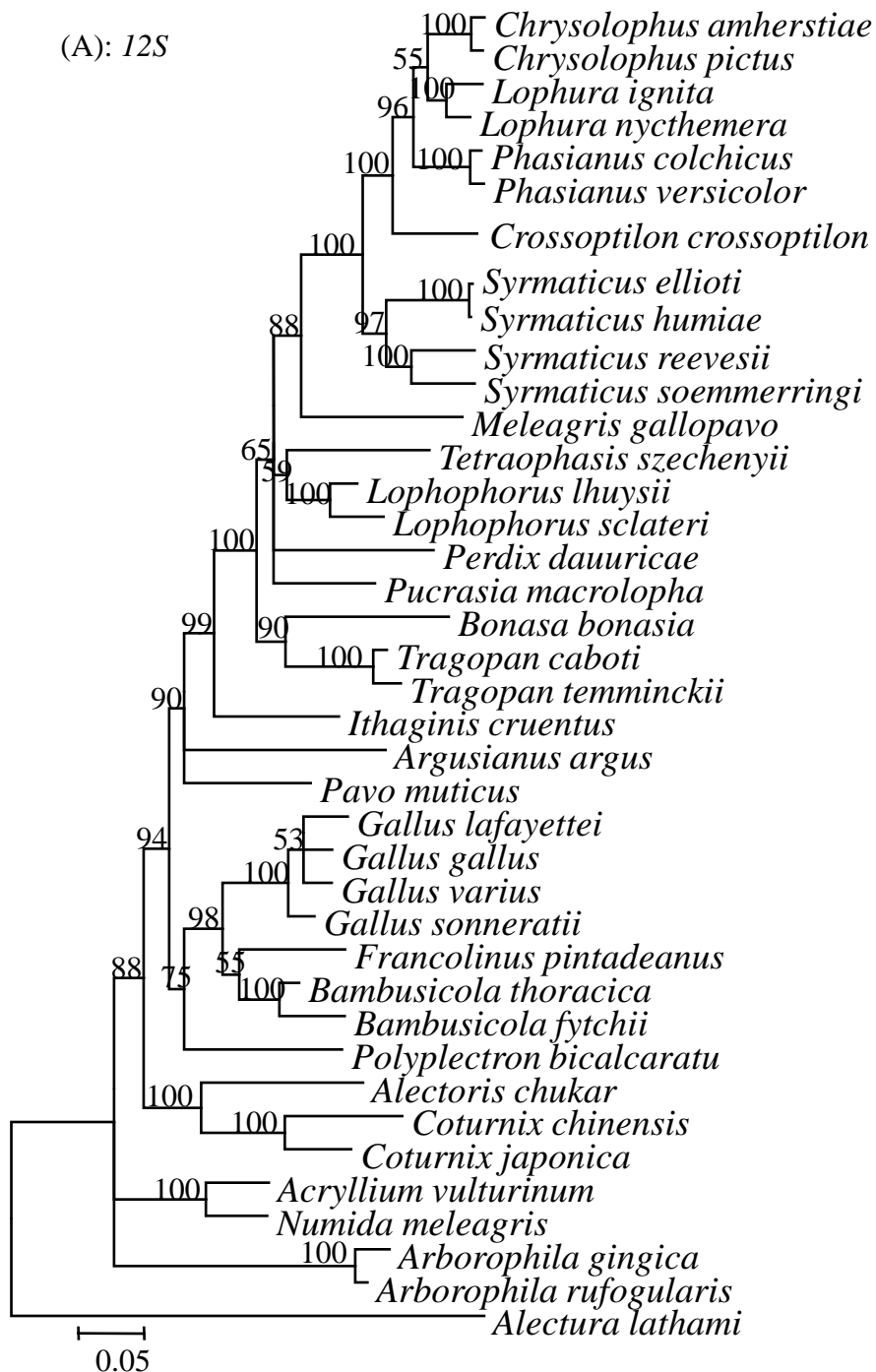

(B): 16S

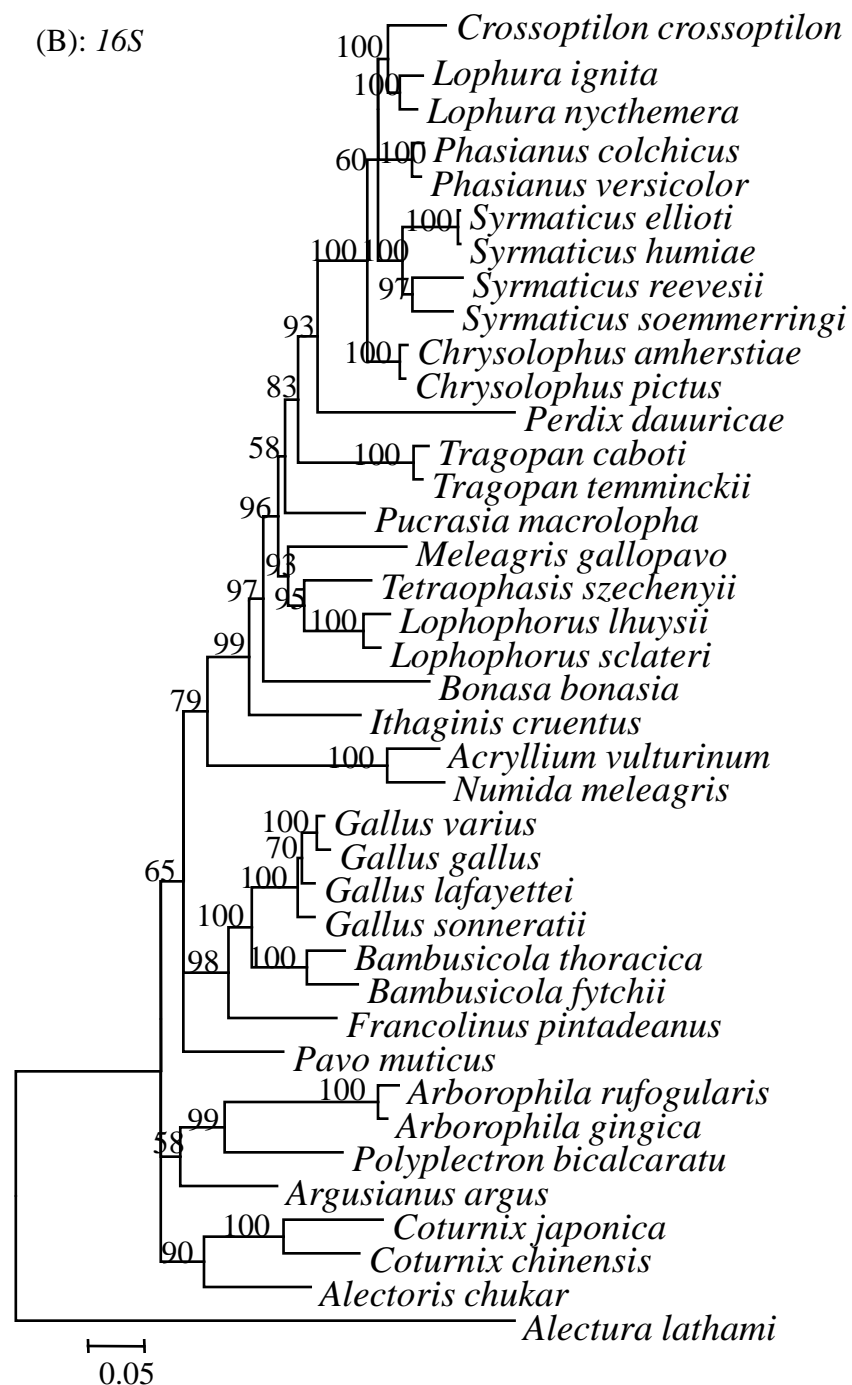

(C): ATP6

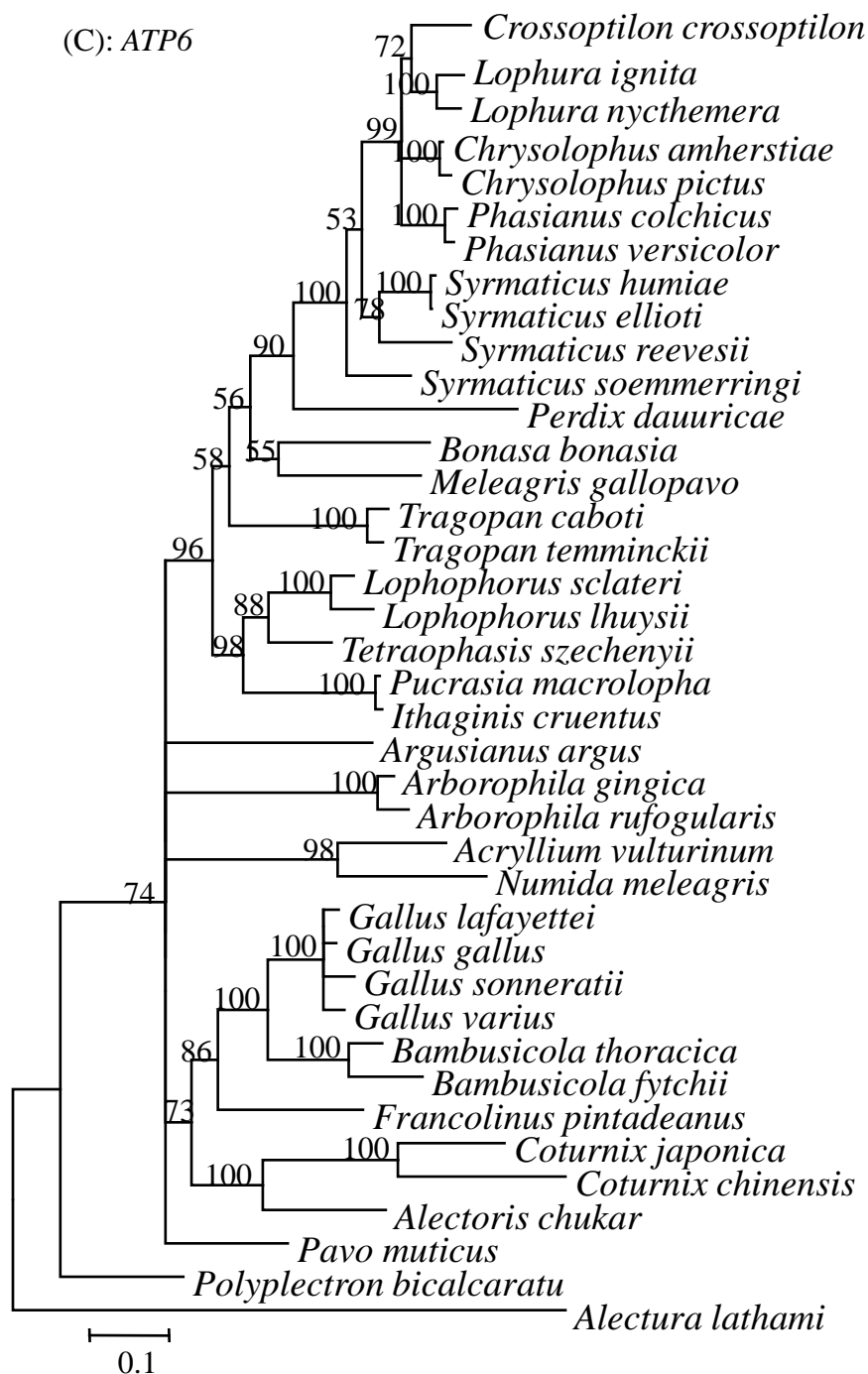

(D): ATP8

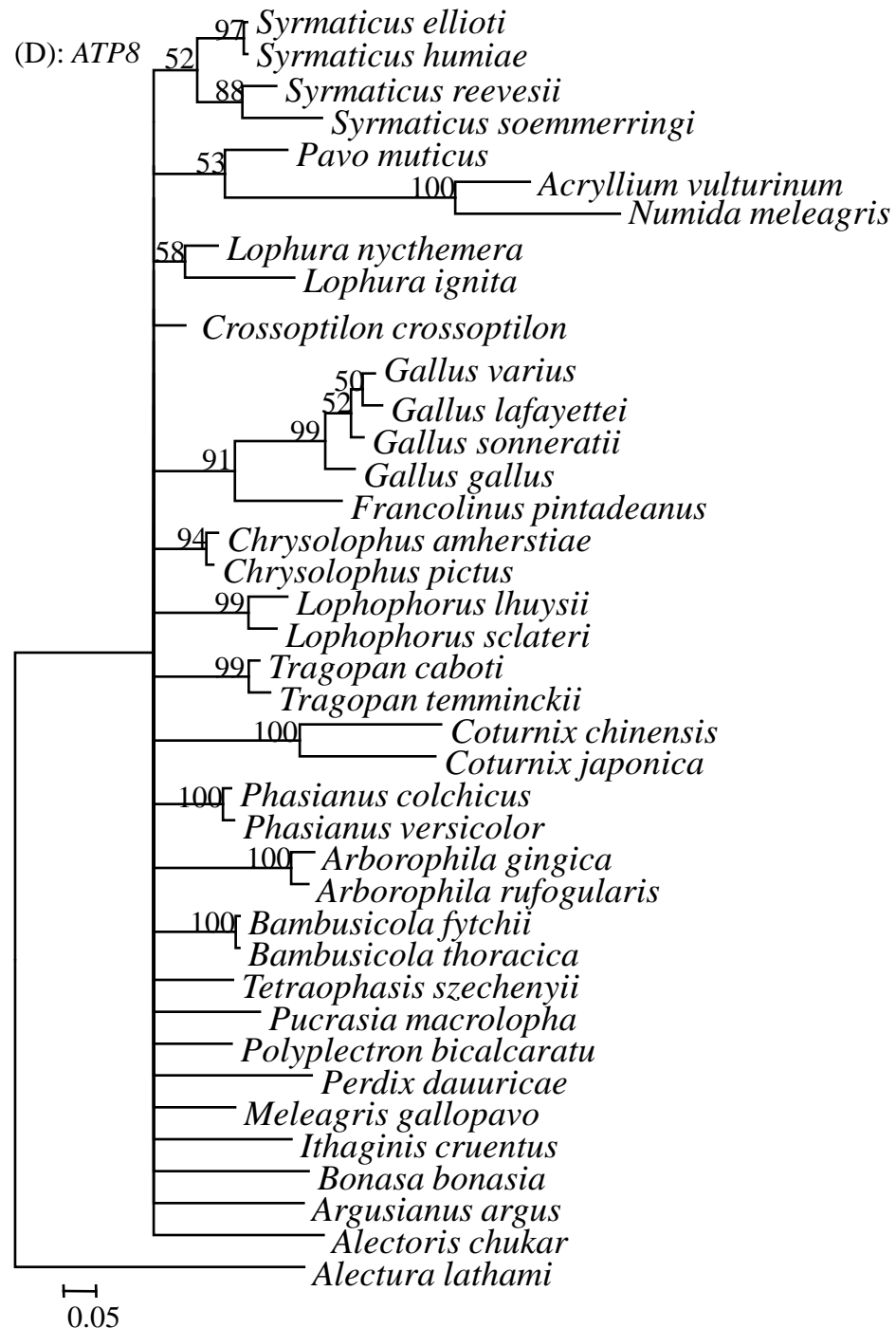

(E): *CoxI*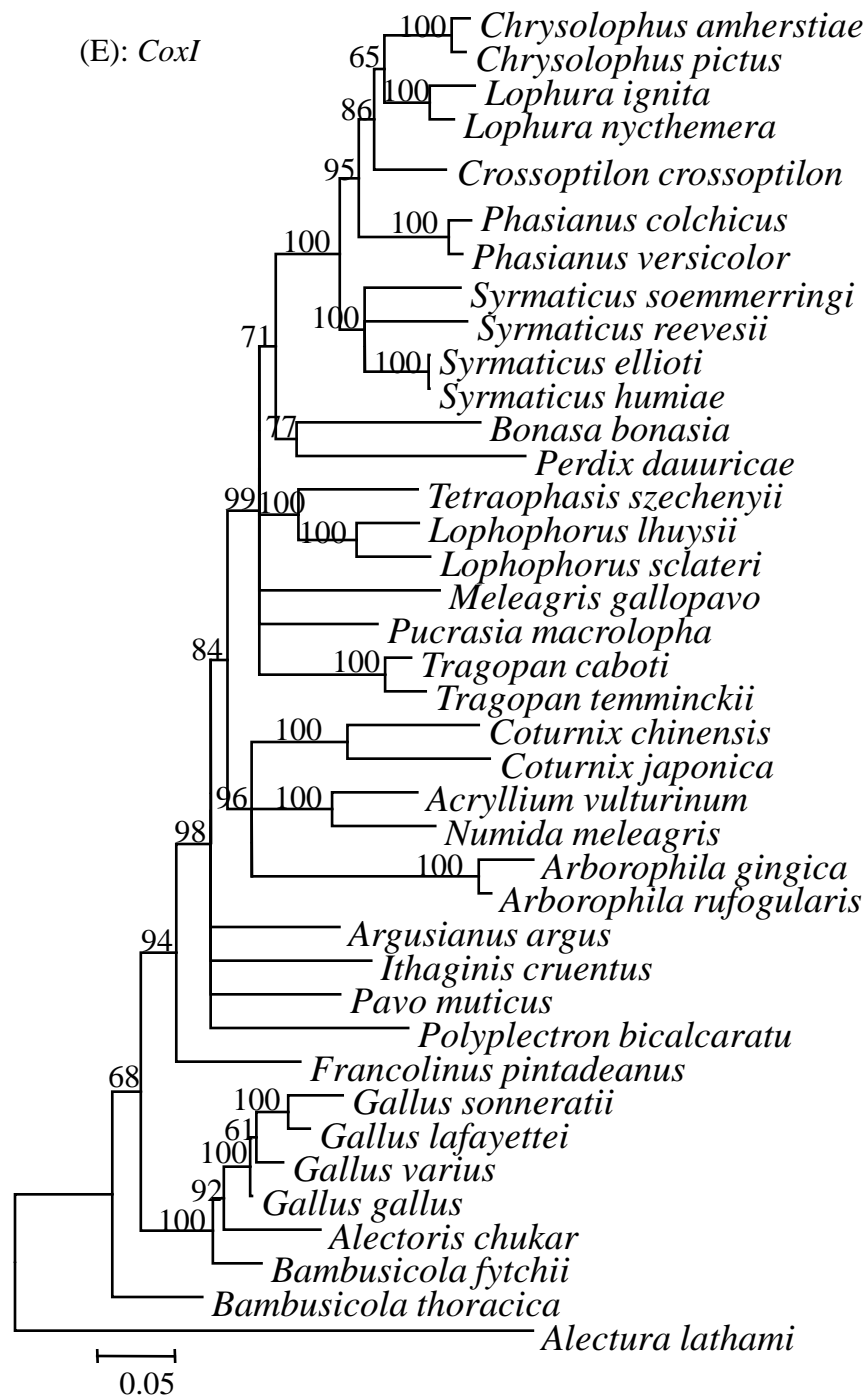(F): *CoxII*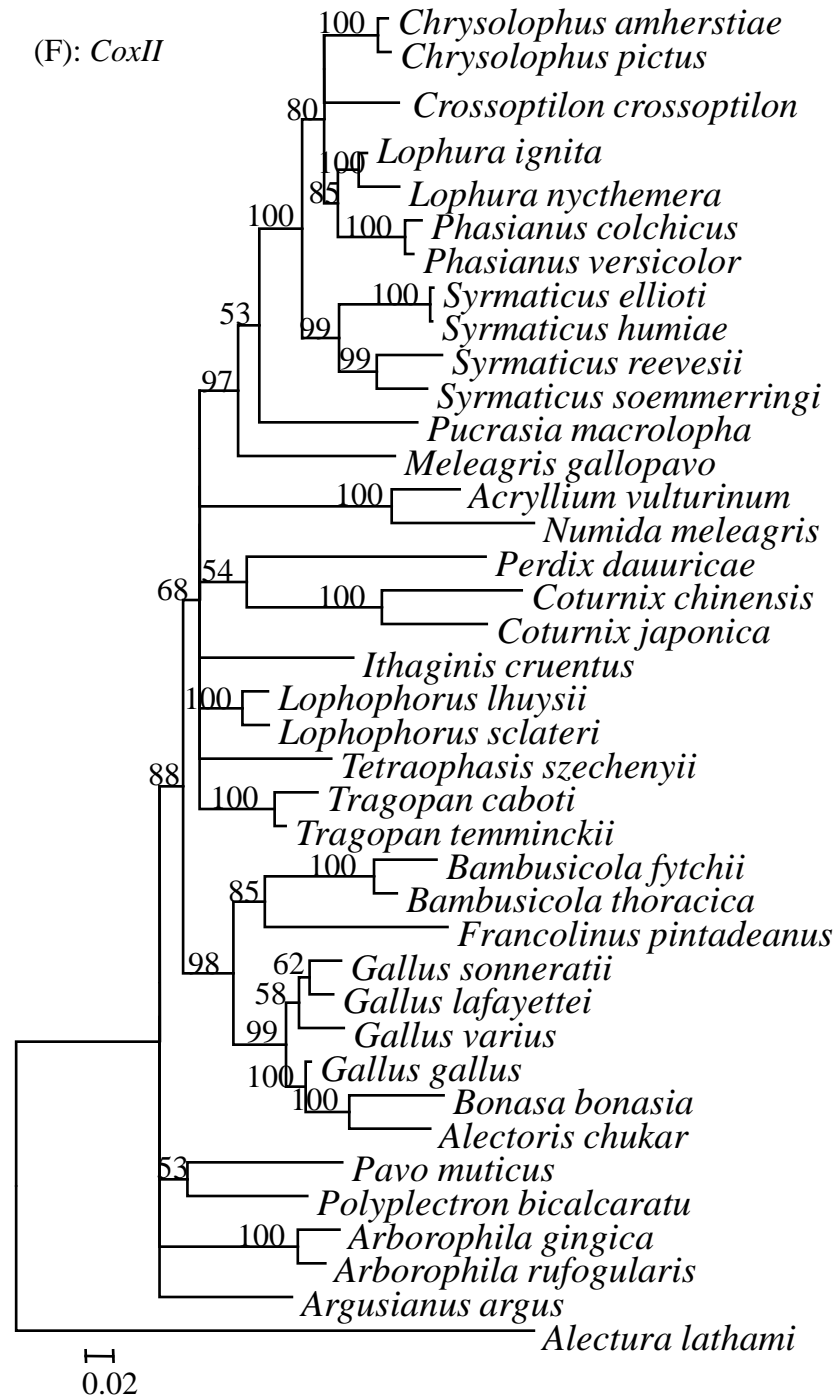

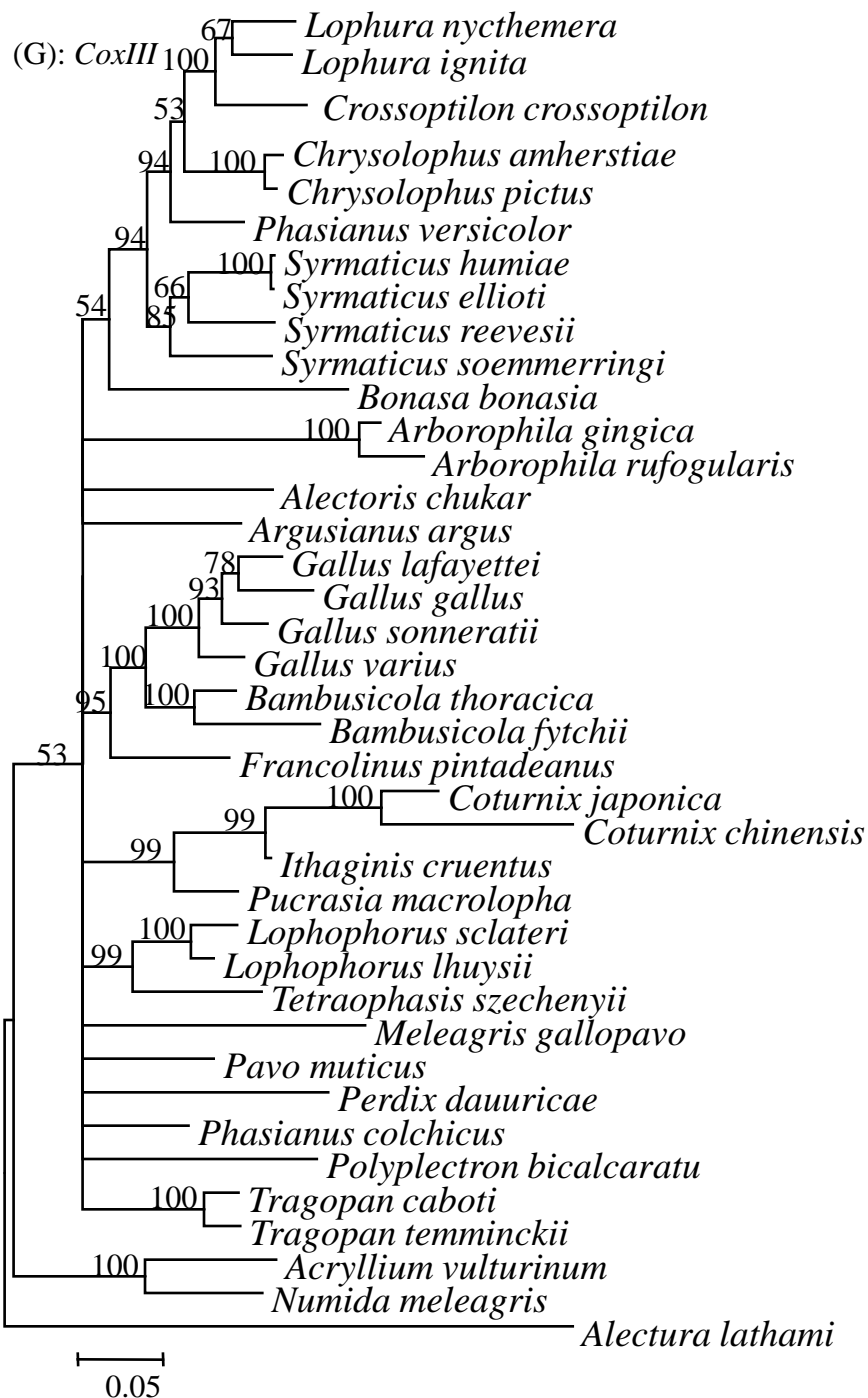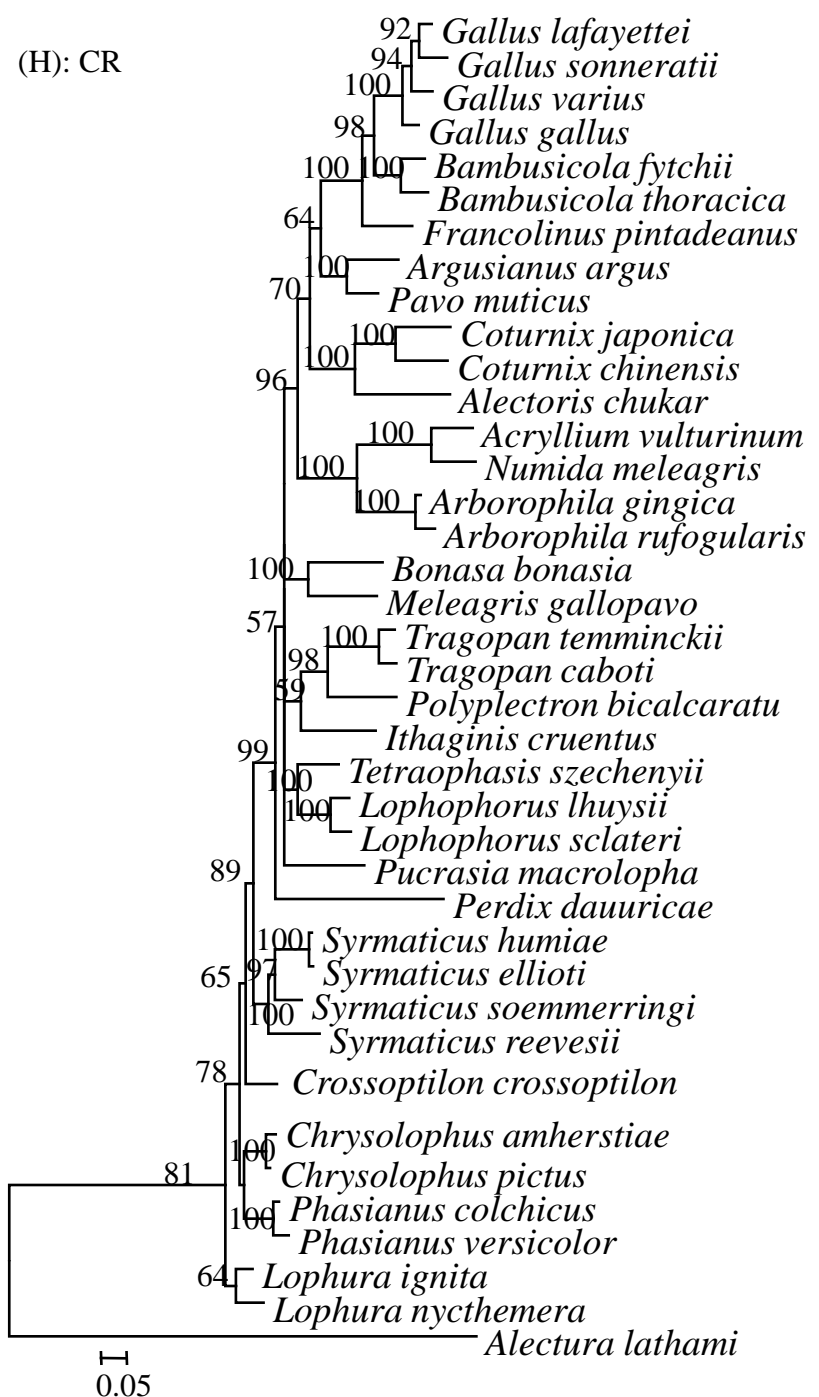

(I): ND1

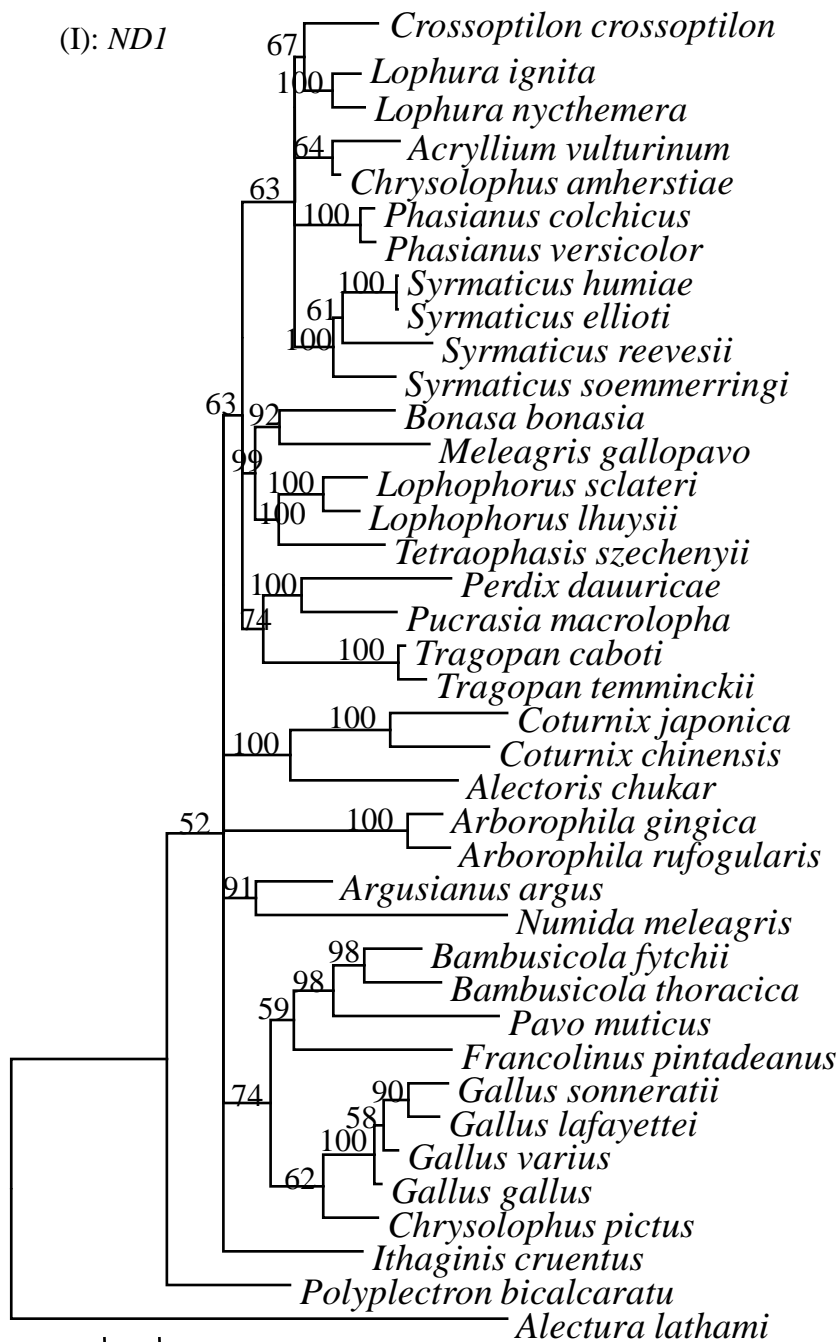

(J): ND2

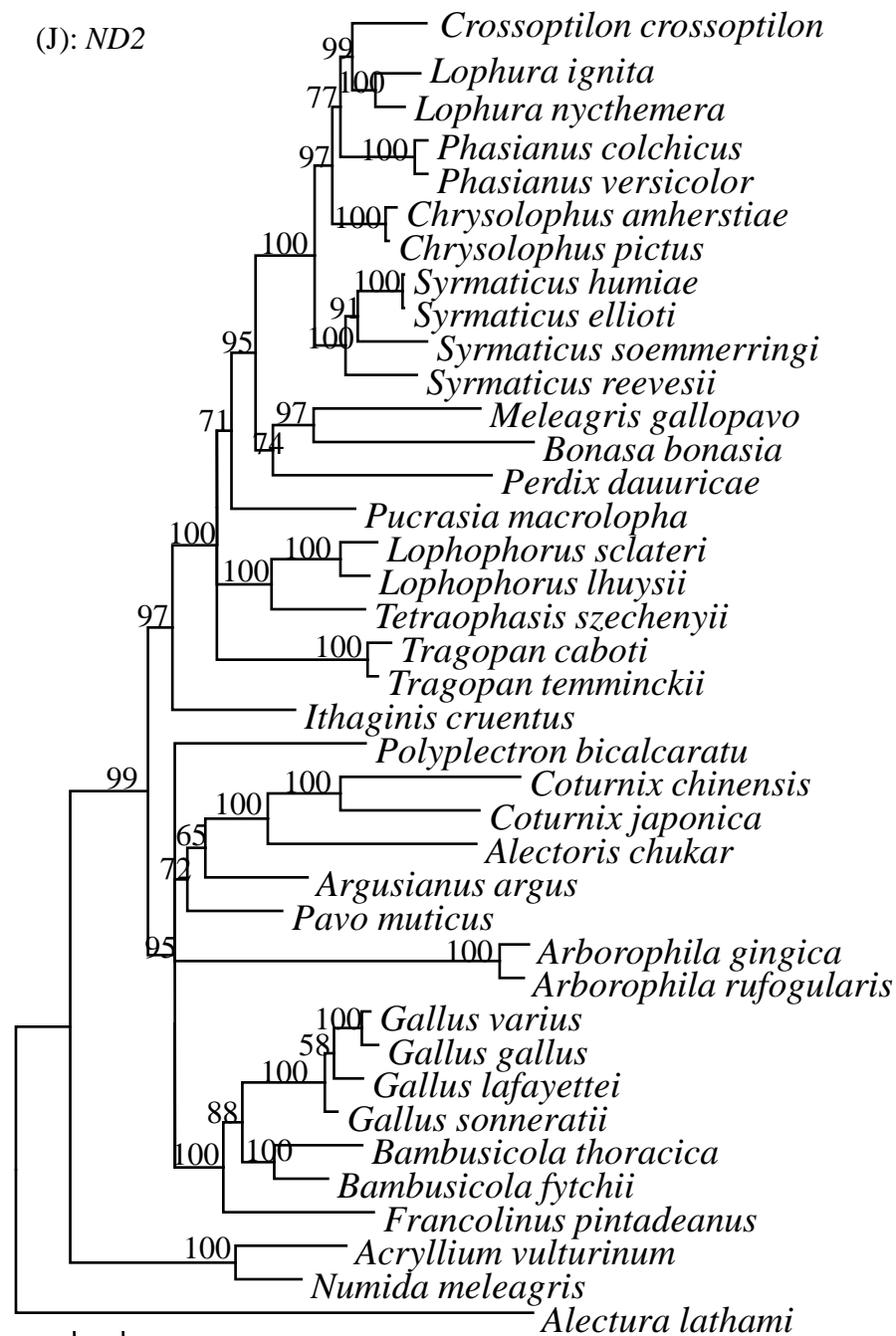

(K): ND3

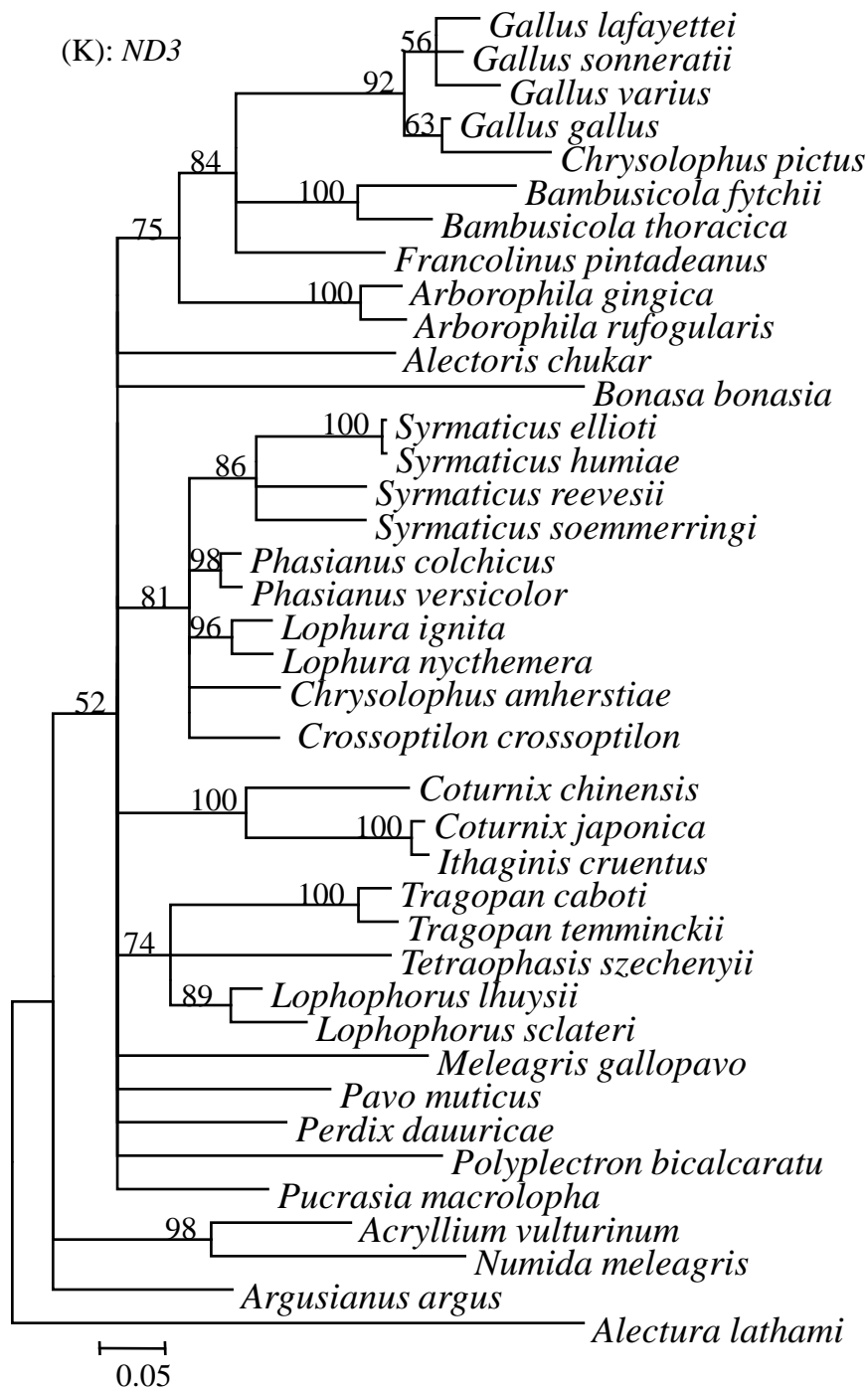

(L): ND4

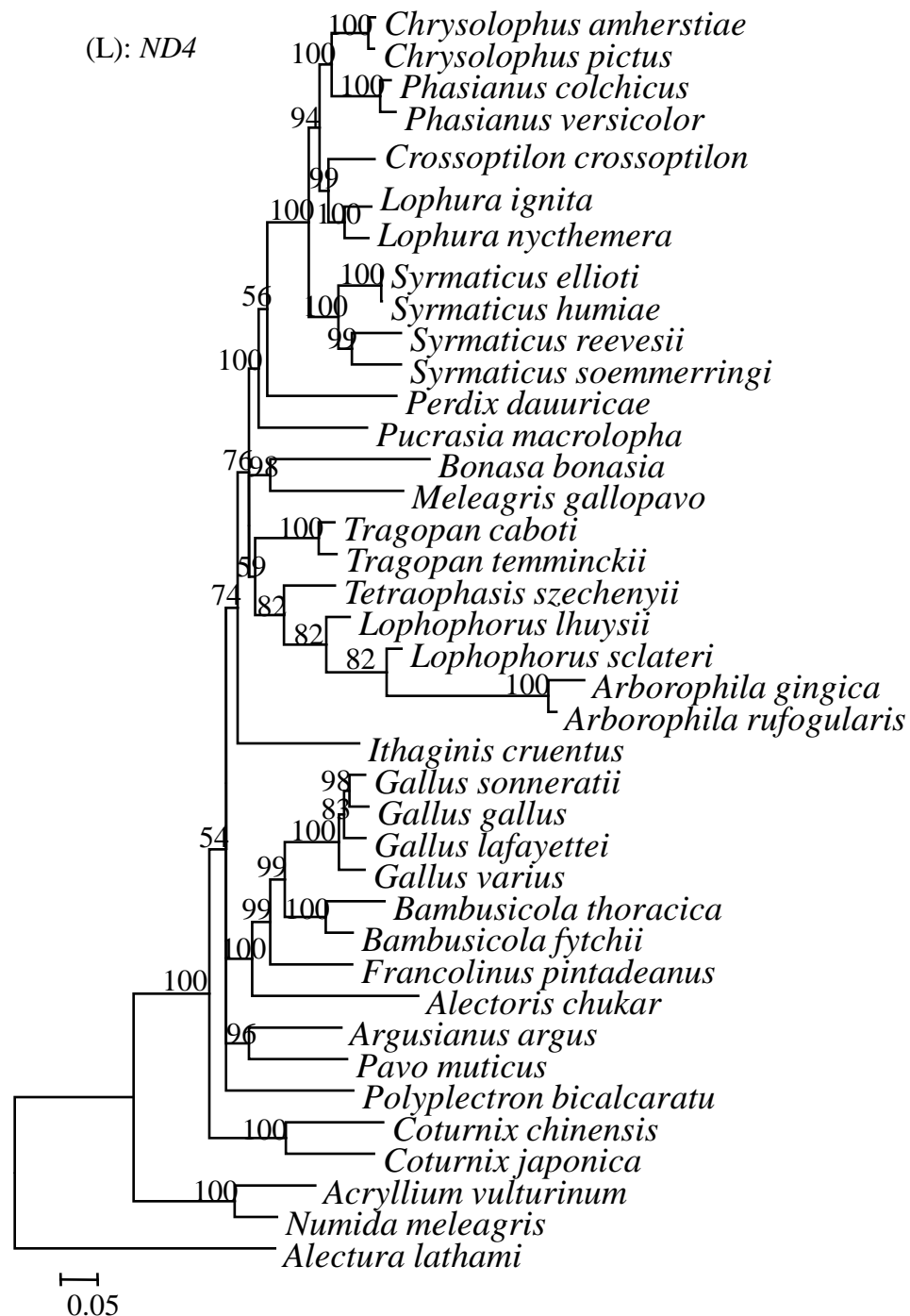

(M): ND4L

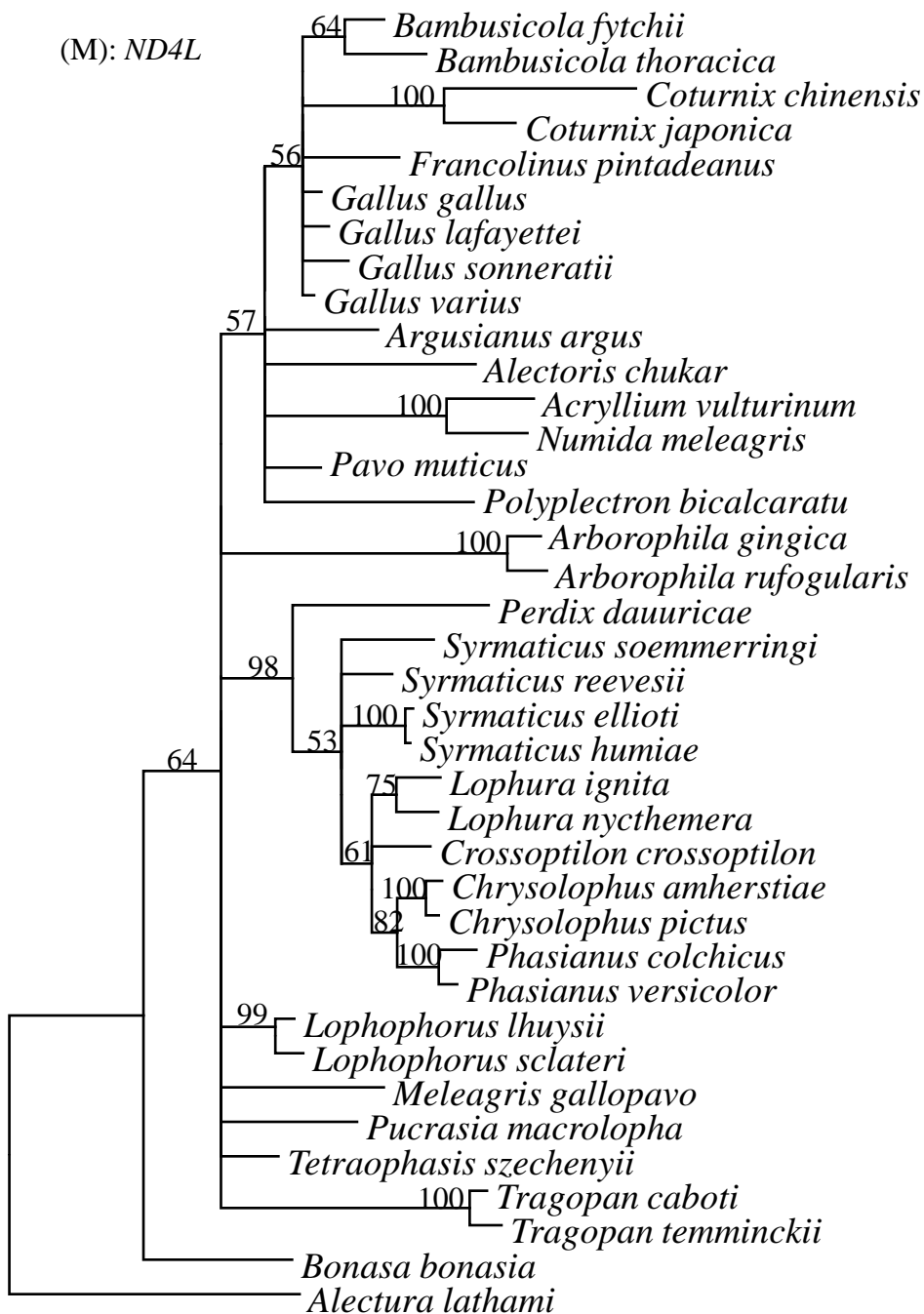

(N): ND5

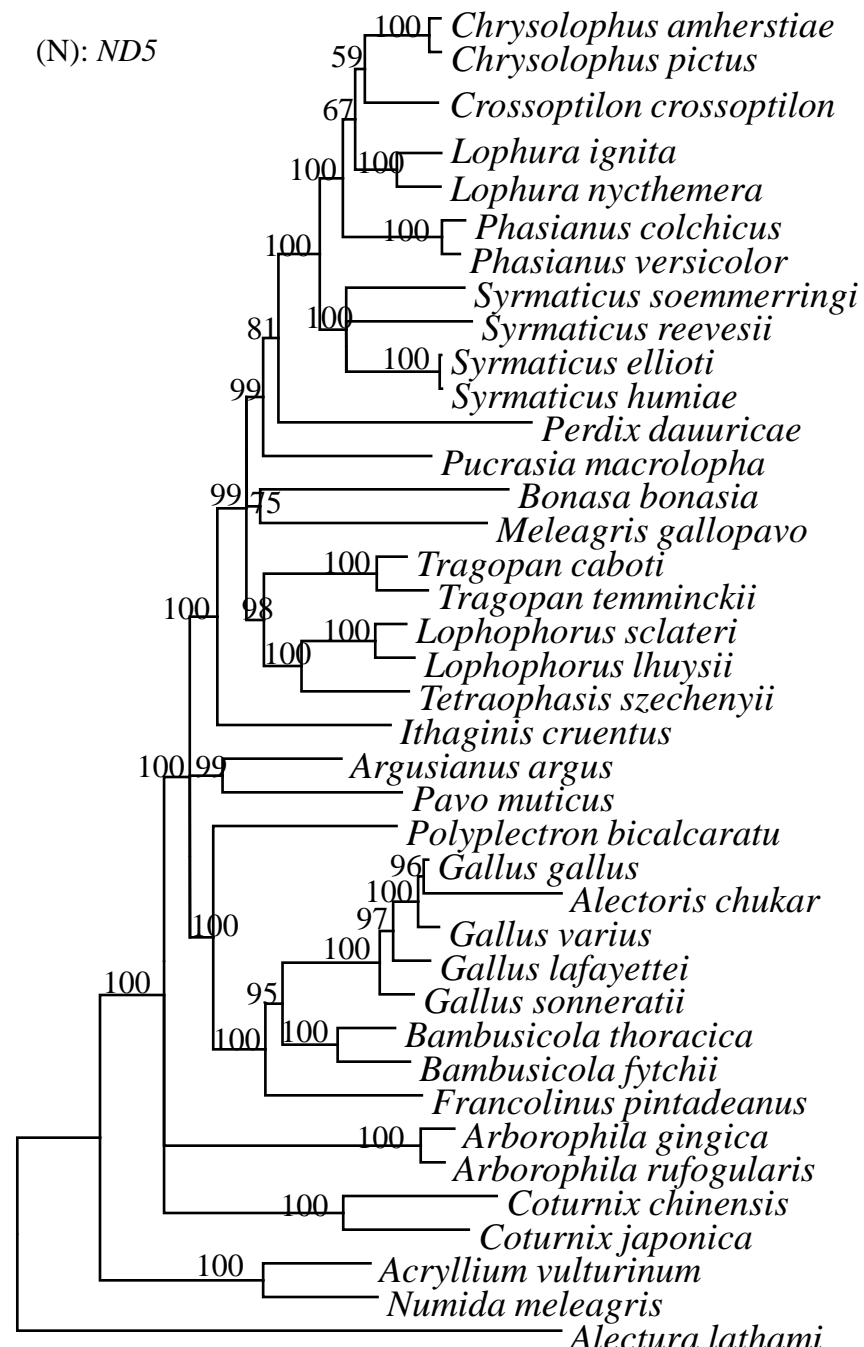

(O): ND6

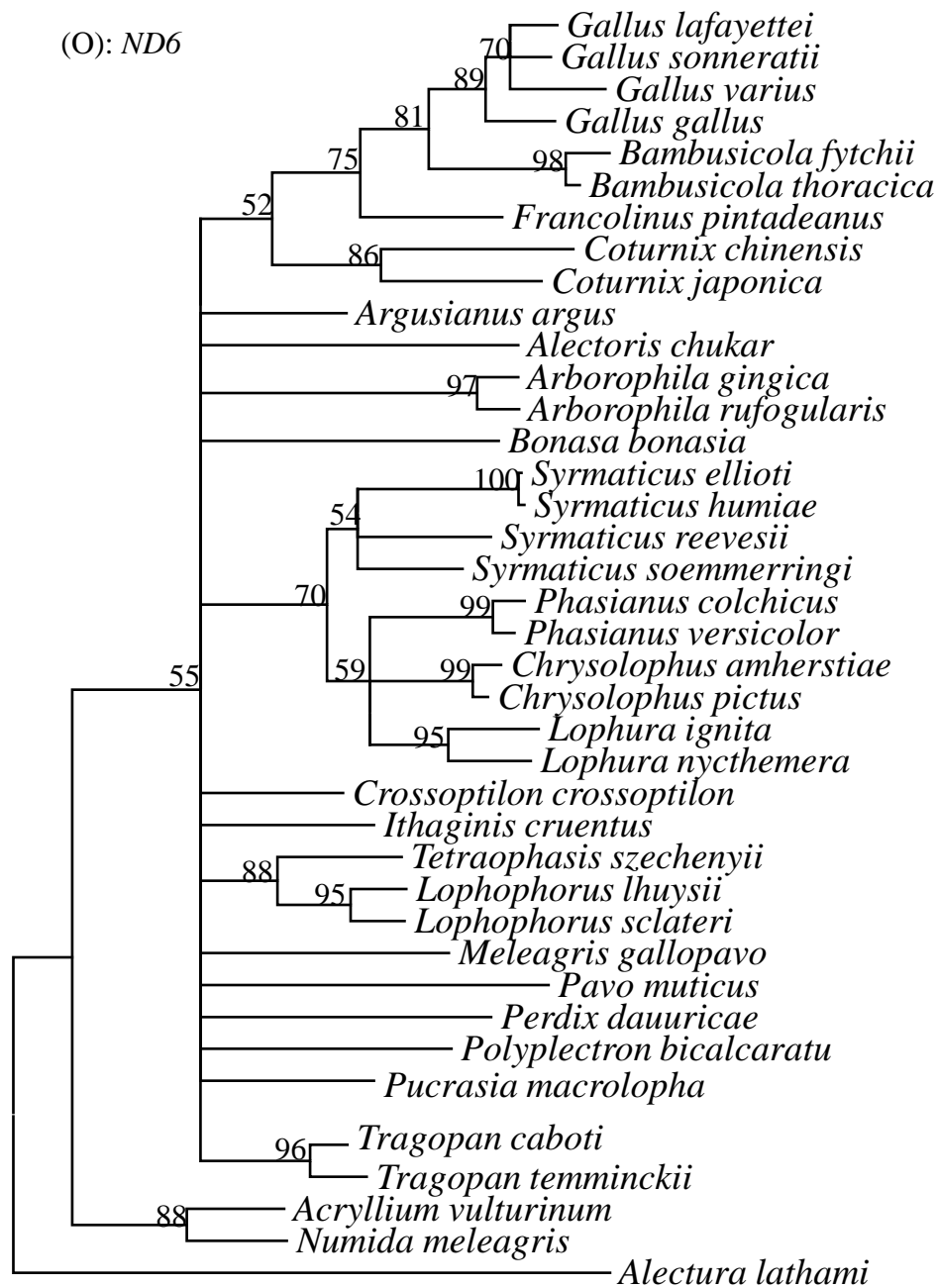

(P): CytB

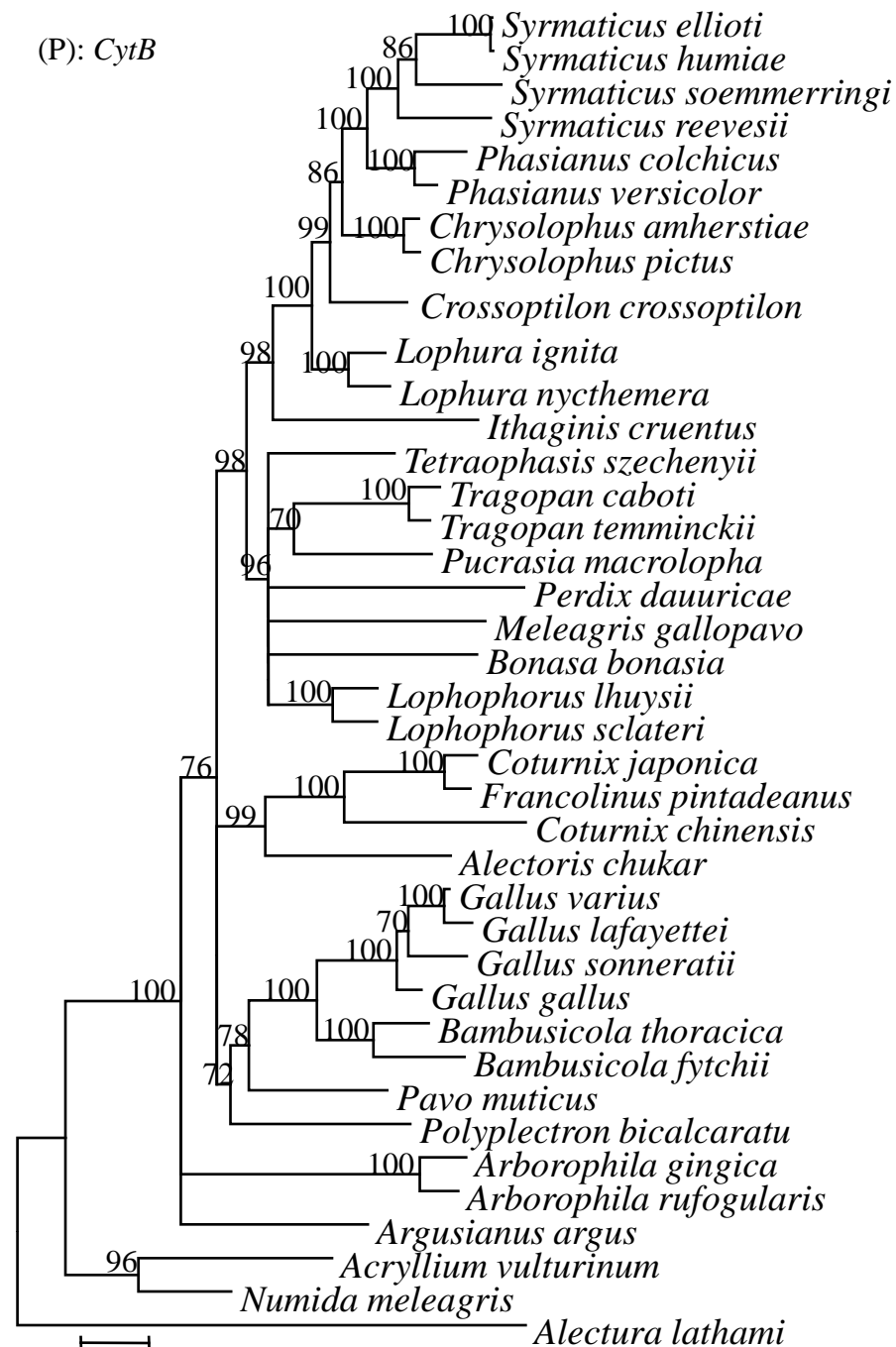

Supplement: Figure S4 — Bayesian inference analyses of individual mt genes and control region (CR). Each run was conducted with 5,000,000 generations and sampled every 100 generations. Bayesian Posterior Probabilities >70% were indicated on the branches. (A) 12S, 1,036 aligned sites; (B) 16S, 1,702 aligned sites;(C) ATP6, 681 aligned sites; (D) ATP8, 165 aligned sites; (E) CoxI, 1,548 aligned sites; (F) CoxII, 681 aligned sites; (G) CoxIII, 783 aligned sites; (H) CR, 1,352 aligned sites; (I) ND1, 972 aligned sites; (J) ND2, 1,038 aligned sites; (K) ND3, 348 aligned sites; (L) ND4, 1,377 aligned sites; (M) ND4L, 291 aligned sites; (N) ND5, 1,818 aligned sites; (O) ND6, 519 aligned sites; (P) CytB, 1,137 aligned sites. (PDF) [file pone.0095786.s004.pdf]
