# Supplementary material for: The Updated Phylogenies of the Phasianidae Based on Combined Data of Nuclear and Mitochondrial DNA
Source: PLoS One. 2014 Apr 18;9(4):e95786. doi: 10.1371/journal.pone.0095786 (PMC3991718; doi:10.1371/journal.pone.0095786)
Supplement: File S1 — This file contains Table S1 and Table S2. Table S1, Source of sequence data for mitochondrial genomes and nuclear segments. Table S2, List of primers used in this study of the Phasianidae. (DOCX) [file pone.0095786.s006.docx]

Table S1: Source of sequence data for mitochondrial genomes and nuclear segments.

| **Species** | **BDNF** | **c-mos** | **FIB4** | **NGFB** | **NTF3** | **OVOG** | **ZENK** | **mitochondrial genome** |
| --- | --- | --- | --- | --- | --- | --- | --- | --- |
| Acryllium vulturinum |  |  | JQ713688 | JQ713709 | JQ713726 | DQ832070 |  | NC_014180 |
| Alectoris chukar | JQ713658 | JQ713671 | JQ713689 | JQ713710 | JQ713727 | AF170987 | JQ713749 | FJ752426 |
| Alectura lathami | EU737955 |  |  |  | EU740240 | DQ832069 |  | NC_007227 |
| Arborophila gingica |  |  |  |  |  |  |  | FJ752425 |
| Arborophila rufogularis | JQ713657 | JQ713672 | JQ713690 | JQ713711 | JQ713728 |  | JQ713750 | FJ752424 |
| Argusianus argus |  |  |  |  |  |  |  | JQ713768* |
| Bambusicola fytchii | JQ713659 | JQ713673 | JQ713691 | JQ713712 | JQ713729 | JQ713745 | JQ713751 | FJ752423 |
| Bambusicola thoracica | JQ713660 | JQ713674 | JQ713692 | JQ713713 | JQ713730 | AF170978 | JQ713752 | NC_011816 |
| Bonasa bonasia | JQ713661 | JQ713675 | JQ713693 | JQ713714 | JQ713731 | JQ713746 | JQ713753 | FJ752435 |
| Chrysolophus amherstiae | JQ713656 | JQ713676 | JQ713694 | JQ713715 | JQ713732 | DQ832080 | JQ713754 | FJ752434 |
| Chrysolophus pictus | JQ713662 | JQ713677 | JQ713695 | JQ713716 | JQ713733 | DQ307014 | JQ713755 | FJ752433 |
| Coturnix chinensis |  |  |  |  |  |  |  | NC_004575 |
| Coturnix japonica | JQ713663 | JQ713678 | JQ713696 | JQ713717 | JQ713734 | AY952773 | JQ713756 | NC_003408 |
| Crossoptilon crossoptilon |  | JQ713679 | JQ713697 |  | JQ713735 |  | JQ713757 | JQ713767* |
| Francolinus pintadeanus | JQ713664 | JQ713680 | JQ713698 | JQ713718 | JQ713736 | JQ713747 |  | NC_011817 |
| Gallus gallus | EU737949 | JQ713681 | JQ713699 | XM_418016 | EU740236 | AF170979 | AF026082 | NC_007236 |
| Gallus lafayettei |  |  |  |  |  |  |  | NC_007239 |
| Gallus sonneratii |  |  |  |  |  |  |  | NC_007240 |
| Gallus varius |  |  |  |  |  |  |  | NC_007238 |
| Ithaginis cruentus |  | JQ713682 | JQ713700 |  |  | DQ832076 |  | JQ713766* |
| Lophophorus lhuysii |  |  |  |  |  |  |  | NC_013979 |
| Lophophorus sclateri | JQ713665 | JQ713683 | JQ713701 | JQ713719 | JQ713737 | JQ713748 | JQ713758 | FJ752432 |
| Lophura ignita |  |  |  |  |  |  |  | NC_010781 |
| Lophura nycthemera | JQ713666 | JQ713684 | JQ713702 |  | JQ713738 | DQ307017 | JQ713759 | NC_012895 |
| Meleagris gallopavo |  |  |  |  |  |  |  | NC_010195 |
| Numida meleagris | EU738039 | U88425 | JQ713703 | EU740159 | EU740321 | AF170975 |  | NC_006382 |
| Pavo muticus | JQ713667 | JQ713685 | JQ713704 | JQ713720 | JQ713739 | AF170989 | JQ713760 | NC_012897 |
| Perdix dauurica | JQ713668 |  | JQ713705 | JQ713721 | JQ713740 |  | JQ713761 | FJ752431 |
| Phasianus colchicus |  | AY447973 | JQ713706 | JQ713722 | JQ713741 | AY952774 | JQ713762 | FJ752430 |
| Phasianus versicolor |  |  |  |  |  |  |  | NC_010778 |
| Polyplectron bicalcaratum | JQ713669 |  | JQ713707 | JQ713723 | JQ713742 | AF331959 | JQ713763 | NC_012900 |
| Pucrasia macrolopha | JQ713670 | JQ713686 | JQ713708 | JQ713724 | JQ713743 | AF170983 | JQ713764 | FJ752429 |
| Syrmaticus ellioti |  |  |  |  |  |  |  | NC_010771 |
| Syrmaticus humiae |  |  |  |  |  |  |  | NC_010774 |
| Syrmaticus reevesii |  |  |  |  |  |  |  | NC_010770 |
| Syrmaticus soemmerringi |  |  |  |  |  |  |  | NC_010767 |
| Tetraophasis szechenyii |  |  |  | JQ713725 | JQ713744 |  | JQ713765 | FJ752428 |
| Tragopan caboti |  |  |  |  |  |  |  | NC_013619 |
| Tragopan temminckii |  |  |  |  |  |  |  | FJ752427 |

Table S2: List of primers used in this study of the Phasianidae.

| Gene |  | **Primer sequences** |
| --- | --- | --- |
| *BDNF* | brain-derived neurotrophic factor | F5’-3’CAGTTCCACCAAGTTAGAA |
|  |  | R5’-3’CTGAATAATTTACCCTGTTATT |
| *CMOS* | oocyte maturation factor Mos | F5’-3’GCCTGGTGCTCCATCGACTGG |
|  |  | R5’-3’GCAAATGAGTAGATGTCTGCT |
| *FIB4* | beta-fibrinogen gene, intron 4 | F5’-3’CTGTAATATCCCGGTGGTTTCAGG |
|  |  | R5’-3’ATTTCAGATGTTTCACCTCCCTTTC |
| *NGFB* | beta-nerve growth factor precursor gene, (NGFB) | F5’-3’GGATGCCAGATGGAACAGAAG |
|  |  | R5’-3’CGCATCAATCCCTCGGCACCC |
| *NTF3* | neurotrophin 3 | F5’-3’ATATTTCTTGCATATCTTCGTT |
|  |  | R5’-3’TATCCATCTCCAGCCTACAAGG |
| *OVOG[*[*1*](#_ENREF_1)*]* | ovomucoid gene, intron G | F5’-3’CAAGACATACGGCAACAARTG |
|  |  | R5’-3’GGCTTAAAGTGAGAGTCCCRTT |
| *ZENK* | zinc finger protein | F5’-3’ATCAAGCCCAGCAGGATGAGGAA |
|  |  | R5’-3’ATGCTCCTGTTCCAGAAGGTGAT |

1. Armstrong, M.H., E.L. Braun, and R.T. Kimball, *Phylogenetic utility of avian ovomucoid intron G: A comparison of nuclear and mitochondrial phylogenies in Galliformes.* Auk, 2001. **118**(3): p. 799-804.
